# A Novel Belief Entropy for Measuring Uncertainty in Dempster-Shafer Evidence Theory Framework Based on Plausibility Transformation and Weighted Hartley Entropy

**DOI:** 10.3390/e21020163

**Published:** 2019-02-10

**Authors:** Qian Pan, Deyun Zhou, Yongchuan Tang, Xiaoyang Li, Jichuan Huang

**Affiliations:** 1 School of Electronics and Information, Northwestern Polytechnical University, Xi’an 710072, China; 2 First Military Representative Office of Air Force Equipment Department, People’s Liberation Army Air Force, Chengdu 610013, China

**Keywords:** Dempster-Shafer evidence theory, uncertainty of basic probability assignment, belief entropy, plausibility transformation, weighted Hartley entropy, Shannon entropy

## Abstract

Dempster-Shafer evidence theory (DST) has shown its great advantages to tackle uncertainty in a wide variety of applications. However, how to quantify the information-based uncertainty of basic probability assignment (BPA) with belief entropy in DST framework is still an open issue. The main work of this study is to define a new belief entropy for measuring uncertainty of BPA. The proposed belief entropy has two components. The first component is based on the summation of the probability mass function (PMF) of single events contained in each BPA, which are obtained using plausibility transformation. The second component is the same as the weighted Hartley entropy. The two components could effectively measure the discord uncertainty and non-specificity uncertainty found in DST framework, respectively. The proposed belief entropy is proved to satisfy the majority of the desired properties for an uncertainty measure in DST framework. In addition, when BPA is probability distribution, the proposed method could degrade to Shannon entropy. The feasibility and superiority of the new belief entropy is verified according to the results of numerical experiments.

## 1. Introduction

Dempster-Shafer evidence theory (DST) [1,2], which was initially introduced by Dempster in the context of statistical inference and then extended by Shafer into a general framework, has drawn great and continued attention in recent years [3,4,5,6]. The DST could be regarded as an extension of probability theory (PT). In DST, the probabilities are assigned to basic probability assignments (BPAs), which is presented to generalize the BPA in probability distribution in PT. The DST has shown its effectiveness and advantages in wide applications with uncertainty in terms of decision making, such as knowledge reasoning [7,8,9], sensor fusion [10,11,12,13], reliability analysis [14,15], fault diagnosis [16,17,18], assessment and evaluation [19,20,21], image recognition [22,23], and others [24,25,26].

Decision making in the framework of DST is based on the combination results of BPAs. Nonetheless, how to measure the uncertainty of BPA is still an open issue, which has not been completely solved [27]. The uncertainty of BPA mainly contains discord uncertainty and non-specificity uncertainty. Working out the uncertainty of BPA is the groundwork and precondition of applying DST to applications [28]. Entropy was initially proposed to measure the uncertainty in statistical thermodynamics [29]. Then Claude Shannon extended this concept to solve the problem of information theory, namely Shannon entropy [30]. Although the Shannon entropy is admitted as an efficient way for measuring uncertainty in PT framework, it is unavailable to be used directly in the DST as the BPA described by sets of probabilities rather than single events [31]. For the sake of better standardizing the uncertainty measure in the framework of DST, Klir and Wierman defined a list of five basic required properties that an uncertainty measure should verify in DST [32]. Many attempts have been made to extend the Shannon entropy for measuring the uncertainty of BPA in the framework of DST, including Dubois and Prade’s weighted Hartley entropy [33], Höhle’s confusion uncertainty measure [34], Yager’s dissonance uncertainty measure [35], Klir and Ramer’s discord uncertainty measure [36], Klir and Parviz’s strife uncertainty measure [37], Jousselme’s ambiguity uncertainty measure [38], and Deng entropy [39]. Generally speaking, these approaches could degenerate to Shannon entropy if the probability values are assigned to single events. A belief entropy following Deng entropy is proposed by Pan and Deng to measure uncertainty in DST [40]. The method borrows from the idea of Deng entropy and is based on the probability interval, which is composed of the belief function and plausibility function. Although this Deng entropy-based method contains more information and could effectively measure the uncertainty in numerical cases, it does not satisfy most of the desired properties. Moreover, the expression of discord uncertainty measure in the method just considers the central values of the lower and upper bounds of the interval, which lacks explicit practical significance. Recently, Jiroušek and Shenoy added four new properties to the set of basic requirements. Thereafter, they define a belief entropy, which could verify six desired properties [41]. Their approach uses the probability mass function (PMF) transformed by plausibility transformation and weighted Hartley entropy to measure the discord and non-specificity uncertainty, respectively. However, the PMF used in the discord uncertainty measure may cause information loss when it is converted from BPA [42]. Hence, the discord uncertainty measure used in Jiroušek and Shenoy’s belief entropy needs to be improved.

In this study, inspired by Pan and Deng’s uncertainty measure [32] and Jiroušek and Shenoy’s uncertainty measure [41], a novel belief entropy is proposed to measure the uncertainty in DST framework. The novel belief entropy has two components, the discord uncertainty measure and non-specificity uncertainty measure. The non-specificity uncertainty measure is the same as Dubois and Prade’s weighted Hartley entropy, which could efficiently reflect the scale of each BPA. The discord uncertainty measure is based on the sum of PMFs transformed by plausibility transformation of single events, which are contained in each BPA. The sum of PMFs could be seen as the representative of probability interval with a practical significance. The discord uncertainty measure in the proposed method could capture sufficient information. In addition, the proposed method could satisfy six basic required properties.

The rest of this study is organized as follows. In Section 2, the preliminaries of DST, probability transformation of BPA, and Shannon entropy are briefly introduced. In Section 3, we discuss the desired properties of uncertainty measure in DST framework. Section 4 presents the exiting belief entropies and the proposed belief entropy. The property analysis of the proposed belief entropy is also conducted in this section. In Section 5, some significant numerical experiments are carried out to illustrate the feasibility and effectiveness of the proposed belief entropy. Finally, in Section 6, the conclusion and future work are summarized.

## 2. Preliminaries

Some basic concepts are briefly introduced in this section, including Dempster-Shafer evidence theory [1,2], probability transformation of transforming a BPA to a PMF [43,44], and Shannon entropy [30].

### 2.1. Dempster-Shafer Evidence Theory

Let $\Theta =\left\{x_{1},x_{2},\ldots ,x_{n}\right\}$ be a nonempty finite set of mutually exclusive and collectively exhaustive alternatives. The Θ is called the frame of discernment (FOD). The power set of Θ is denoted by $2^{\Theta }$, namely

(1) $$2^{\Theta }=\left\{\emptyset ,\left\{x_{1}\right\},\left\{x_{2}\right\},\ldots ,\left\{x_{n}\right\},\left\{x_{1},x_{2}\right\},\ldots ,\left\{x_{1},x_{2},\ldots ,x_{i}\right\},\ldots ,\Theta \right\},$$

A BPA is a mapping *m* from power set $2^{\Theta }$ to $\left[0,1\right]$, which satisfies the condition:

(2) $$m\left(\emptyset \right)=0and\sum_{A\in 2^{\Theta }}m(A)=1.$$

*A* is called a focal element such that m(A)>0. The BPA is also known as mass function.

There are two functions associated with each BPA called belief function Bel(A) and plausibility function Pl(A), respectively. The two functions are defined as follows:

(3) $$\begin{array}{c} Bel\left(A\right)=\sum_{B\subseteq A}m(A),\\ Pl\left(A\right)=\sum_{A\cap B\neq \emptyset }m(A). \end{array}$$

The plausibility function Pl(A) denotes the degree of BPA that potentially supports *A*, while the belief function Bel(A) denotes the degree of BPA that definitely supports *A*. Thus, Bel(A) and Pl(A) could be seen as the lower and upper probability of *A*.

Suppose $m_{1}$ and $m_{2}$ are two independent BPAs in the same FOD Θ, and they can be combined by using the Dempster-Shafer combination rule as follows:

(4) $$m\left(A\right)=m_{1}⊕m_{2}=\left\{\begin{array}{c} \frac{\sum_{B\cap C=A}m_{1}\left(B\right)m_{2}\left(C\right)}{1-k},A\neq \emptyset \\ 0,A=\emptyset \end{array},\right.$$

with

(5) $$k=\sum_{B\cap C=\emptyset }m_{1}\left(B\right)m_{2}\left(C\right),$$

where the *k* is the conflict coefficient to measure the degree of conflict among BPAs. The operator ⊕ denotes the Dempster-Shafer combination rule. Please note that the Dempster-Shafer combination rule is unavailable for combining BPAs such that k>0.

### 2.2. Probability Transformation

There are many ways to transform a BPA *m* to a PMF. Here, the pignistic transformation and the plausibility transformation are introduced.

Let *m* be a BPA on FOD Θ. Its associated probabilistic expression of PMF on Θ is defined as follows:

(6) $$BetP\left(x\right)=\sum_{A\in 2^{\Theta },x\in A}\frac{m(A)}{\left|A\right|},$$

where the $\left|A\right|$ is the cardinality of *A*. The transformation between *m* and BetP(x) is called the pignistic transformation.

Pt(x) is a probabilistic expression of PMF that is obtained from *m* by using plausibility transformation as follows:

(7) $$Pt\left(x\right)=\frac{Pl(x)}{\sum_{x\in \Theta }Pl(x)},$$

where the Pl(x) is the plausibility function of specific element *x* in Θ. The transformation between *m* and Pt(x) is called the plausibility transformation.

### 2.3. Shannon Entropy

Let Ω be a FOD with possible values $\left\{w_{1},w_{2},\ldots ,w_{n}\right\}$. The Shannon entropy is explicitly defined as:

(8) $$H_{s}=\sum_{w_{i}\in \Omega }p\left(w_{i}\right)\log_{2}\left[\frac{1}{p\left(w_{i}\right)}\right],$$

where the $p\left(w_{i}\right)$ is the probability of alternative $w_{i}$, which satisfies $\sum_{i=1}^{n}p\left(w_{i}\right)=1$. If some $p\left(w_{i}\right)=0$, we follow the convention that $p\left(w_{i}\right)\log_{2}\left[\frac{1}{p\left(w_{i}\right)}\right]=0$ as $\lim_{x\to 0^{+}}x\log_{2}\left(x\right)=0$. Please note that we will simply use log for $\log_{2}$ in the rest of this paper.

## 3. Desired Properties of Uncertainty Measures in The DS Theory

In the research of Klir and Wierman [32], Klir and Lewis [45], and Klir [46], five basic required properties are defined for uncertainty measure in DST framework, namely probabilistic consistency, set consistency, range, sub-additivity, and additivity. These requirements are detailed as follows.

- *Probability consistency*. Let *m* be a BPA on FOD *X*. If *m* is a Bayesian BPA, then $H\left(m\right)=\sum_{x\in X}m\left(x\right)\log\left[\frac{1}{m(x)}\right]$.
- *Additivity*. Let $m_{X}$ and $m_{Y}$ be distinct BPAs for FOD *X* and FOD *Y*, respectively. The combined BPA $m_{X}⊕m_{Y}$ using Dempster-Shafer combination rules must satisfy the following equality:
  (9) $$H\left(m_{X}⊕m_{Y}\right)=H\left(m_{X}\right)+H\left(m_{Y}\right),$$
  where the $m_{X}⊕m_{Y}$ is a BPA for $\left\{X,Y\right\}$. For all $A\times B\in 2^{\left\{X,Y\right\}}$, where $A\in 2^{X}$ and $B\in 2^{Y}$, we have:
  (10) $$\left(m_{X}⊕m_{Y}\right)\left(A\times B\right)=m_{X}\left(A\right)m_{Y}\left(B\right)$$
- *Sub-additivity*. Let *m* be a BPA on the space X×Y, with marginal BPAs $m^{↓X}$ and $m^{↓Y}$ on FOD *X* and FOD *Y*, respectively. The uncertainty measure must satisfy the following inequality:
  (11) $$H\left(m\right)\leq H\left(m^{↓X}\right)+H\left(m^{↓Y}\right)$$
- *Set consistency*. Let *m* be a BPA on FOD *X*. If there exists a focal element A∈X and m(A)=1, then an uncertainty measure must degrade to Hartley measure:
  (12) $$H\left(m\right)=\log\left|A\right|.$$
- *Range*. Let *m* be a BPA on FOD *X*. The range of an uncertainty measure H(m) must be $\left[0,\log\left|X\right|\right]$.

These properties illuminated in DST framework start from the verification by Shannon entropy in PT. In DST, there exist more situations of uncertainty than in PT framework [47]. Therewith, by analyzing shortcomings of these properties, Jiroušek and Shenoy add four other desired properties for measuring uncertainty in DST framework, including consistency with DST semantics, non-negativity, maximum entropy, monotonicity [41].

The uncertainty measure for BPA in DST must agree on the DST semantics [48]. Many uncertainty measures are based on the PMFs which are transformed from BPA [49,50,51]. However, only the plausibility transformation is compatible with the Dempster-Shafer combination rule [41,44]. Therefore, the property of consistency with DST semantics is presented to require the uncertainty measure to satisfy the tenets in DST framework.

- *Consistency with DST semantics*. Let $m_{1}$ and $m_{2}$ be two BPAs in the same FOD. If an uncertainty measure is based on a probability transformation of BPA, which transforms a BPA *m* to a PMF $P_{m}$, then the PMFs of $m_{1}$ and $m_{2}$ must satisfy the following condition:
  (13) $$P_{m_{1}⊕m_{2}}=P_{m_{1}}⊗P_{m_{2}},$$
  where ⊗ denotes the Bayesian combination rule [41], i.e., pointwise multiplication followed by normalization. Notice that this property is not presupposing the use of probability transformation in the uncertainty measure.

The property of additivity is easy to satisfy by most definitions of uncertainty measure [41]. The property of consistency with DST semantics is regarded as reinforcement of the additivity property, which makes sure that any uncertainty measure in DST framework follows the Dempster-Shafer combination rule.

Since the number of uncertainty type in DST framework is larger than that in PT framework. One can find that uncertainty measures in DST framework prefer a wider range than that in PT framework, namely $\left[0,\log\left|X\right|\right]$. Thus, in Jiroušek and Shenoy’s opinion, the properties of non-negativity, maximum entropy, and monotonicity are pivotal to uncertainty measure in DST framework.

- *Non-negativity*. Let *m* be a BPA on FOD *X*. The uncertainty measure H(m) must satisfy the following inequality:
  (14) H(m)≥0,
  where the equality holds up if and only if *m* is Bayesian and $m\left\{\left(x\right)\right\}=1$ with x∈X.
- *Maximum entropy*. Let *m* be a BPA on FOD *X*. The vacuous BPA $m_{v}$ should have the most uncertainty, then the uncertainty measure must satisfy the following inequality:
  (15) $$H\left(m_{v}\right)\geq H\left(m\right),$$
  where the equality holds up if and only if $m=m_{v}$.
- *Monotonicity*. Let $v_{X}$ and $v_{Y}$ be the vacuous BPAs of FOD *X* and FOD *Y*, respectively. If $\left|X\right|<\left|Y\right|$, then $H\left(v_{X}\right)<H\left(v_{Y}\right)$.

The property of set consistency entails that the uncertainty of a vacuous BPA $m_{v}$ for FOD *X* is $\log\left|X\right|$. The probability consistency entails that the uncertainty of a Bayesian BPA $m_{e}$, which has the equally likely probabilities for *X*, is $\log\left|X\right|$ too. However, these two requirements are contradictory as the property of maximum entropy consider $H\left(m_{v}\right)>H\left(m_{e}\right)$. About this contradiction, there is a debatable open issue. Some researchers suggest the uncertainty of these two kinds of BPA should be equal and be the maximum possible uncertainty as we cannot get information to help us make a determinate decision [52,53]. Some other researchers deem the uncertainty of a vacuous BPA to be greater than a Bayesian uniform BPA, which is demonstrated by Ellsberg paradox phenomenon [54,55,56]. To provide a comprehensive understanding for our definition of uncertainty measure, all the above-mentioned properties are taken into account.

## 4. The Belief Entropy for Uncertainty Measure in DST Framework

### 4.1. The Existing Definitions of Belief Entropy of BPAs

The majority of the uncertainty measures have the Shannon entropy as the start point, which plays an important role to address the uncertainty in PT framework. Nevertheless, the Shannon entropy has inherent limitations to handle the uncertainty in DST as there are more types of uncertainty [27,57]. This is reasonable because the BPA includes more information than probabilistic distribution [4]. In the earlier literatures, the definitions of belief entropy only focus on one aspect of discord uncertainty or non-specificity uncertainty in the BPAs. Then, Yager makes a contribution to distinction between the discord uncertainty and non-specificity uncertainty [35]. Thereafter, the discord and non-specificity are taken into consideration in most of the definitions of belief entropy. Some representative belief entropies and their definitions are listed as follows:

**Höhel**. One of the earliest uncertainty measures in DST is presented by Höhel as shown [34]:

(16) $$H_{o}\left(m\right)=\sum_{A\in 2^{X}}m(A)\log\left[\frac{1}{Bel(A)}\right],$$

where the Bel(A) is the belief function of proposition *A*. $H_{o}\left(m\right)$ only considers the discord uncertainty measure.

**Nguyen** defines the belief entropy of BPA *m* using the original BPAs [58]:

(17) $$H_{n}\left(m\right)=\sum_{A\in 2^{X}}m(A)\log\left[\frac{1}{m(A)}\right].$$

As the definition of $H_{o}\left(m\right)$, $H_{n}\left(m\right)$ only captures the discord part of uncertainty.

**Dubois and Prade** define the belief entropy using the cardinality of BPAs [33]:

(18) $$H_{d}\left(m\right)=\sum_{A\in 2^{X}}m(A)\log\left|A\right|.$$

$H_{d}\left(m\right)$ considers only the non-specificity portion of the uncertainty. Dubois and Prade’s definition could be regarded as the weighted Hartley entropy $H_{h}\left(m\right)$, where $H_{h}\left(m\right)=\log\left|A\right|$.

**Pal et al.** define a belief entropy as [59]:

(19) $$H_{p}\left(m\right)=\sum_{A\in 2^{X}}m(A)\log\left[\frac{1}{m(A)}\right]+\sum_{A\in 2^{X}}m(A)\log\left(\left|A\right|\right).$$

In $H_{p}\left(m\right)$, the first component is the measure of discord uncertainty, and the second component is the measure of non-specificity uncertainty.

**Jousselme et al.** define a belief entropy based on the pignistic transformation [38]:

(20) $$H_{j}\left(m\right)=\sum_{x\in X}BetP(x)\log\left[\frac{1}{BetP(x)}\right],$$

where the BetP(x) is the PMF of pignistic transformation. The $H_{j}\left(m\right)$ using the Shannon entropy of BetP(x)

**Deng** defines a belief entropy, namely Deng entropy, as follows [39]:

(21) $$H_{deng}\left(m\right)=\sum_{A\in 2^{X}}m(A)\log\left[\frac{1}{m(A)}\right]+\sum_{A\in 2^{X}}m(A)\log\left[2^{\left|A\right|}-1\right].$$

The $H_{deng}\left(m\right)$ is very similar to the definition of $H_{p}\left(m\right)$, while $H_{deng}\left(m\right)$ employs the $2^{\left|A\right|}-1$ instead of $\left|A\right|$ to measure the non-specificity uncertainty of the BPA.

**Pan and Deng** develop Deng entropy $H_{deng}\left(m\right)$ with the definition [40]:

(22) $$H_{pd}\left(m\right)=\sum_{A\in 2^{X}}\frac{1}{2}\left[Bel(A)+Pl(A)\right]\log\left\{\frac{1}{\frac{1}{2}\left[Bel(A)+Pl(A)\right]}\right\}+\sum_{A\in 2^{X}}m(A)\log\left[2^{\left|A\right|}-1\right],$$

where the Bel(A) and Pl(A) are the belief function and plausibility function, respectively. $H_{pd}\left(m\right)$ uses the central value of the probability interval $\left[Bel(A),Pl(A)\right]$ to measure the discord uncertainty of BPA.

It is obvious that all these uncertainty measures are the extension of the Shannon entropy in DST. Apart from the aforementioned methods of belief entropy, there are, of course, some other entropy-based uncertainty measures for BPAs in DST framework. One can find an expatiatory and detailed introduction to these methods in the literature [41,47].

**Jiroušek and Shenoy** define a concept for measuring uncertainty, as follows [41]:

(23) $$H_{JS}\left(m\right)=\sum_{x\in X}Pt\left(x\right)\log\left[\frac{1}{Pt(x)}\right]+\sum_{A\in 2^{X}}m(A)\log\left(\left|A\right|\right).$$

The $H_{JS}\left(m\right)$ consists of two components. The first part is Shannon entropy of a PMF based on the plausibility transformation, which is associated with discord uncertainty. The second part is the entropy of Dubois and Prade for measuring non-specificity in BPAs. The $H_{JS}\left(m\right)$ satisfies the six desired properties, including consistency with DST semantics, non-negativity, maximum entropy, monotonicity, probability consistency, and additivity. Moreover, the properties of range and set consistency are expanded.

### 4.2. The Proposed Belief Entropy

Although the $H_{JS}\left(m\right)$ can better meet the requirement of the basic properties for uncertainty measure, it has an intrinsic defect. The first part in $H_{JS}\left(m\right)$ using Shannon entropy captures only the probability of plausibility transformation, which may lead to information loss. As argued in $H_{pd}\left(m\right)$, the probability interval $\left[Bel(A),Pl(A)\right]$ can provide more information according to the BPAs in each proposition. However, the $H_{pd}\left(m\right)$ considers only the numerical average of the probability interval, which lacks the piratical physical significance. In this study, by combining the merit of $H_{JS}\left(m\right)$ and $H_{pd}\left(m\right)$, a new definition of belief entropy-based uncertainty measure in DST framework is proposed as follows:

(24) $$H_{PQ}\left(m\right)=\sum_{A\in 2^{X}}m(A)\log\left[\frac{1}{Pm(A)}\right]+\sum_{A\in 2^{X}}m(A)\log\left(\left|A\right|\right),$$

where the $Pm\left(A\right)=\sum_{x\in A}Pt(x)$ is the summation of plausibility transformation-based PMFs of *x* contained in *A*.

Similar to most of the belief entropies, the first component $\sum_{A\in 2^{X}}m\left(A\right)\log\left[Pm^{-1}\left(A\right)\right]$ in $H_{PQ}\left(m\right)$ is designed to measure the discord uncertainty of BPA. The information contained in not only BPAs but also the plausibility function based on Pt(x) is taken into consideration. Since the Pt(x) reflects the support degree of different propositions to element *x*, it could provide more information than m(A). Furthermore, the $Pm\left(A\right)=\sum_{x\in A}Pt(x)$ satisfies the Bel(A)≤Pm(A)≤Pl(A), which could be seen as a representative of the probability interval. At length, the second component $\sum_{A\in 2^{X}}m\left(A\right)\log\left(\left|m(A)\right|\right)$ in $H_{PQ}$ is the same as the $H_{d}\left(m\right)$ to measure the non-specificity uncertainty of BPA. Therefore, we believe that the new proposed belief entropy can be more effective to measure the uncertainty of BPAs in DST framework. The property analysis of $H_{PQ}\left(m\right)$ is explored as follows.

(1) Consistency with DST semantics. The first part in $H_{PQ}\left(m\right)$ uses Pt(x) based on the plausibility transformation, which is compatible with the definition of the property. The second part is not a Shannon entropy based on probability transformation. Thus, $H_{PQ}\left(m\right)$ satisfies the consistency with DST semantics property.

(2) Non-negativity. As Pm(A)∈[0,1], m(A)∈[0,1] and $0<\left|m(A)\right|$, thus, $H_{PQ}\left(m\right)\geq 0$. If and only if the *m* is a Bayesian BPA and m(x)=1, $H_{PQ}\left(m\right)=0$. Thus, $H_{PQ}\left(m\right)$ satisfies the non-negativity property.

(3) Maximum entropy. Let $m_{e}$ and $m_{v}$ be a uniform Bayesian BPA and a vacuous BPA in the same FOD *X*, respectively. We could obtain $H_{PQ}\left(m_{e}\right)=H_{PQ}\left(m_{v}\right)=\log\left(\left|X\right|\right)$, therefore $H_{PQ}\left(m\right)$ dissatisfies the maximum entropy property.

(4) Monotonicity. Since $H_{PQ}\left(m_{v}\right)=\log\left(\left|X\right|\right)$, $H_{PQ}\left(m_{v}\right)$ is monotonic in |X|. Therefore $H_{PQ}\left(m\right)$ satisfies the monotonicity property.

(5) Probability consistency. If *m* is a Bayesian BPA, then Pm(x)=Pt(x)=m(x) and $H_{d}\left(m\right)=0$. Hence, $H_{PQ}\left(m\right)=\sum_{x\in X}m\left(x\right)\log\left[\frac{1}{m(x)}\right]$. Therewith, we know that the $H_{PQ}\left(m\right)$ satisfies the probability consistency property.

(6) Set consistency. If a focal element has the whole support degree such that m(A)=1, $H_{PQ}\left(m\right)=\log\left(\left|X\right|\right)$. Hence, the $H_{PQ}\left(m\right)$ satisfies the set consistency property.

(7) Range. As Pm(A) includes the support from the other propositions, thus, m(A)≤Pm(A). Therefore, $\sum_{A\in 2^{X}}m\left(A\right)\log\left[Pm^{-1}\left(A\right)\right]\leq \sum_{A\in 2^{X}}m\left(A\right)\log\left[m^{-1}\left(A\right)\right]=H_{n}\left(m\right)$. The range of $H_{n}\left(m\right)$ and $H_{d}\left(m\right)$ both are $\left[0,\log\left(\left|X\right|\right)\right]$. Thus, the range of $H_{PQ}\left(m\right)$ is $\left[0,2\log\left(\left|X\right|\right)\right]$, which means the $H_{PQ}\left(m\right)$ dissatisfies the range property.

(8) Additivity. Let $m_{X}$ and $m_{Y}$ be two BPAs of FOD *X* and FOD *Y*, respectively, $A\subseteq 2^{X}$, and $B\subseteq 2^{Y}$. Let C=A×B be the corresponding joint focal element on X×Y, x∈X, and y∈Y. Let *m* be a joint BPA defined on X×Y which is obtained by using Equation (10). Thus, $m\left(C\right)=\left(m_{X}⊕m_{Y}\right)\left(A\times B\right)=m_{X}\left(A\right)m_{Y}\left(B\right)$. Then the new belief entropy for *m* is:

$$H_{PQ}\left(m\right)=H_{PQ}\left(m_{X}⊕m_{Y}\right)=\sum_{C\in 2^{\left\{X\times Y\right\}}}m(C)\log\left[\frac{\left|C\right|}{Pm(C)}\right],$$

where

$$\begin{array}{c} m\left(C\right)=m_{X}\left(A\right)m_{Y}\left(B\right), \end{array}$$

$$Pm\left(C\right)=Pm\left(A\times B\right)=\sum_{\left(x,y\right)\in \left\{A\times B\right\}}Pt(x,y)=\sum_{\left(x,y\right)\in \left\{A\times B\right\}}\frac{Pl(x,y)}{\sum_{\left(x,y\right)\in \left\{X\times Y\right\}}Pl(x,y)}.$$

As proved in [33], we have $Pl\left(A\times B\right)=Pl\left(A\right)Pl\left(B\right)$. Thus, we know

$$\sum_{\left(x,y\right)\in \left\{A\times B\right\}}\frac{Pl\left(x,y\right)}{\sum_{\left(x,y\right)\in \left\{X\times Y\right\}}Pl\left(x,y\right)}=\sum_{x\in A}\sum_{y\in B}\frac{Pl\left(x\right)Pl\left(y\right)}{\sum_{x\in X}\sum_{y\in Y}Pl\left(x\right)Pl\left(y\right)}=\sum_{x\in A}\frac{Pl(x)}{\sum_{x\in X}Pl(x)}\sum_{y\in B}\frac{Pl\left(y\right)}{\sum_{y\in Y}Pl\left(y\right)}$$

and

$$Pm\left(C\right)=\sum_{x\in A}\frac{Pl(x)}{\sum_{x\in X}Pl(x)}\sum_{y\in B}\frac{Pl\left(y\right)}{\sum_{y\in Y}Pl\left(y\right)}=Pm\left(A\right)Pm\left(B\right).$$

Consequently,

$$\begin{array}{cc} H_{PQ}\left(m_{X}⊕m_{Y}\right) & =\sum_{C\in 2^{\left\{X\times Y\right\}}}m\left(C\right)\log\left[\frac{\left|A\right|\left|B\right|}{Pm\left(A\right)Pm\left(B\right)}\right]\\ & =\sum_{A\in 2^{\left\{X\right\}}}\sum_{B\in 2^{\left\{Y\right\}}}m_{X}\left(A\right)m_{Y}\left(B\right)\log\left[\frac{\left|A\right|}{Pm(A)}\right]+\sum_{A\in 2^{\left\{X\right\}}}\sum_{B\in 2^{\left\{Y\right\}}}m_{X}\left(A\right)m_{Y}\left(B\right)\log\left[\frac{\left|B\right|}{Pm\left(B\right)}\right]\\ & =\sum_{A\in 2^{\left\{X\right\}}}m_{X}\left(A\right)\sum_{B\in 2^{\left\{Y\right\}}}m_{Y}\left(B\right)\log\left[\frac{\left|A\right|}{Pm(A)}\right]+\sum_{A\in 2^{\left\{X\right\}}}m_{X}\left(A\right)\sum_{B\in 2^{\left\{Y\right\}}}m_{Y}\left(B\right)\log\left[\frac{\left|B\right|}{Pm\left(B\right)}\right]\\ & =\sum_{A\in 2^{\left\{X\right\}}}m_{X}\left(A\right)\log\left[\frac{\left|A\right|}{Pm(A)}\right]+\sum_{B\in 2^{\left\{Y\right\}}}m_{Y}\left(B\right)\log\left[\frac{\left|B\right|}{Pm\left(B\right)}\right]\\ & =H_{PQ}\left(m_{X}\right)+H_{PQ}\left(m_{Y}\right). \end{array}$$

Hence, the $H_{PQ}\left(m\right)$ satisfies the additivity property.

(9) Sub-additivity. An example of binary-valued variables is given to check whether the $H_{PQ}\left(m\right)$ satisfies the sub-additivity as follows with masses

$$m\left(z_{11}\right)=m\left(z_{12}\right)=0.1,m\left(z_{21}\right)=m\left(z_{22}\right)=0.3,m\left(X\times Y\right)=0.2,$$

where $z_{ij}=\left(x_{i},y_{j}\right)$. The marginal BPAs of for *X* and *Y* are $m^{↓X}$ and $m^{↓Y}$, respectively, shown as following ones.

$$\begin{array}{c} m^{↓X}\left(x_{1}\right)=0.2,m^{↓X}\left(x_{2}\right)=0.6,m^{↓X}\left(X\right)=0.2\\ m^{↓Y}\left(y_{1}\right)=0.4,m^{↓Y}\left(y_{2}\right)=0.4,m^{↓Y}\left(Y\right)=0.2 \end{array}$$

Thus,

$$\begin{array}{c} Pl\left(x_{1}\right)=0.4,Pl\left(x_{2}\right)=0.8,Pl\left(y_{1}\right)=0.5,Pl\left(y_{2}\right)=0.5,\\ Pl\left(Z_{11}\right)=0.3,Pl\left(Z_{12}\right)=0.3,Pl\left(Z_{21}\right)=0.5,Pl\left(Z_{22}\right)=0.5\\ Pt\left(x_{1}\right)=0.333,Pt\left(x_{2}\right)=0.667,Pt\left(y_{1}\right)=0.5,Pt\left(y_{2}\right)=0.5,\\ Pt\left(Z_{11}\right)=0.188,Pt\left(Z_{12}\right)=0.188,Pt\left(Z_{21}\right)=0.312,Pt\left(Z_{22}\right)=0.312\\ H_{PQ}\left(m\right)=1.8899,H_{PQ}\left(m^{↓X}\right)+H_{PQ}\left(m^{↓Y}\right)=0.8678+1=1.8687. \end{array}$$

Obviously, $H_{PQ}\left(m\right)>H_{PQ}\left(m^{↓X}\right)+H_{PQ}\left(m^{↓Y}\right)$, thus the $H_{PQ}\left(m\right)$ dissatisfies the sub-additivity property.

In summary, the new belief entropy $H_{PQ}\left(m\right)$ for uncertainty measure in DST framework satisfies the properties of consistency with DST semantics, non-negativity, set consistency, probability consistency, additivity, monotonicity, and does not satisfy the properties of sub-additivity, maximum entropy, range. An overview of the properties of existing belief entropies for uncertainty measure are listed in Table 1.

Additionally, based on combining the advantages of the definition of Jiroušek-Shenoy and Pan-Deng, the new belief entropy involves more information, which can better meet the requirements. The properties of maximum entropy and range that the new belief entropy dissatisfies need further discussion. For maximum entropy properties, we think that the uncertainty of a vacuous BPA and an equally likely Bayesian BPA should be equivalent. There is a classical example.

Assume a bet on a race conducted by four cars, *A*, *B*, *C*, and *D*. Two experts give their opinion. Expert-1 suggests that the ability of the four drivers and the performance of the four cars are almost the same. Expert-2 has no idea about the traits of each car and driver. The opinion of the Expert-1 could be regarded as a uniform probability distribution with $m\left(A\right)=m\left(B\right)=m\left(C\right)=m\left(D\right)=\frac{1}{4}$. while the Expert-2 produces a vacuous BPA with m(A,B,C,D)=1. Based on only one piece of these two pieces of evidence, we have no information to support us to make a certain bet. Besides, it is very convincing that the range property is not suitable for uncertain measure. The range $\left[0,\log\left(\left|X\right|\right)\right]$ can only reflect one aspect of uncertainty, which lacks consideration for multiple uncertainties of a BPA in DST framework. As a consequence, the properties of maximum entropy and range should be extended.

## 5. Numerical Experiment

In this section, several numerical experiments are verified to demonstrate the reasonability and effectiveness of our proposed new belief entropy.

### 5.1. Example 1

Let $\Theta =\left\{x\right\}$ be the FOD. Given a BPA with m(x)=1, we can obtain the Pt(x) and Pm(x) with:

Pt(x)=1,Pm(x)=1.

Then, the associated Shannon entropy $H_{s}\left(m\right)$ and the proposed belief entropy $H_{PQ}\left(m\right)$ are calculated as follows:

$$H_{s}\left(m\right)=1\times \log\left(1\right)=0,H_{PQ}\left(m\right)=1\times \log\left(\frac{1}{1}\right)=0.$$

Obviously, the above example shows that the Shannon entropy and the proposed belief entropy are equal when the FOD has only one single element, where exits no uncertainty.

### 5.2. Example 2

Let $\Theta =\left\{x_{1},x_{2},x_{3},x_{4},x_{5}\right\}$ be the FOD. A uniform BPA of FOD is given as $m\left(x_{1}\right)=m\left(x_{2}\right)=m\left(x_{3}\right)=m\left(x_{4}\right)=m\left(x_{5}\right)=\frac{1}{5}$. Then,

$$\begin{array}{c} Pt\left(x_{1}\right)=Pt\left(x_{2}\right)=Pt\left(x_{3}\right)=Pt\left(x_{4}\right)=Pt\left(x_{5}\right)=\frac{1}{5},\\ Pm\left(x_{1}\right)=Pm\left(x_{2}\right)=Pm\left(x_{3}\right)=Pm\left(x_{4}\right)=Pm\left(x_{5}\right)=\frac{1}{5},\\ H_{s}\left(m\right)=\frac{1}{5}\times \log5+\frac{1}{5}\times \log5+\frac{1}{5}\times \log5+\frac{1}{5}\times \log5+\frac{1}{5}\times \log5=2.3219,\\ H_{PQ}\left(m\right)=\frac{1}{5}\times \log5+\frac{1}{5}\times \log5+\frac{1}{5}\times \log5+\frac{1}{5}\times \log5+\frac{1}{5}\times \log5+\ldots \\ \ldots +\frac{1}{5}\times \log1+\frac{1}{5}\times \log1+\frac{1}{5}\times \log1+\frac{1}{5}\times \log1+\frac{1}{5}\times \log1=2.3219. \end{array}$$

As shown above, the proposed belief entropy is the same as the Shannon entropy when the BPA is the probability distribution. Section 5.1 and Section 5.2 verify that the proposed belief entropy will degenerate into the Shannon entropy when the belief is assigned to singleton elements.

### 5.3. Example 3

Let $\Theta =\left\{x_{1},x_{2},x_{3},x_{4},x_{5}\right\}$ be the FOD. A vacuous BPA of FOD is given as $m(x_{1},x_{2},x_{3},x_{4},x_{5})=1$. Then,

$$\begin{array}{c} Pt\left(x_{1}\right)=Pt\left(x_{2}\right)=Pt\left(x_{3}\right)=Pt\left(x_{4}\right)=Pt\left(x_{5}\right)=\frac{1}{5},\\ Pm\left(x_{1}\right)=Pm\left(x_{2}\right)=Pm\left(x_{3}\right)=Pm\left(x_{4}\right)=Pm\left(x_{5}\right)=\frac{1}{5},\\ H_{PQ}\left(m\right)=1\times \log1+1\times \log5=2.3219. \end{array}$$

Compared to Section 5.2, we know that the uncertainty of this example is the same as the Section 5.2. This is reasonable. As discussed in Section 4.2, neither the uniform BPA nor the vacuous BPA in the same FOD could provide more information for a determinate single element. Thus, their uncertainty should be equal.

### 5.4. Example 4

Two experiments in [40] are recalled in this example. Let $\Theta =\left\{x_{1},x_{2},x_{3},x_{4}\right\}$ be the FOD. Two BPAs are given as $m_{1}$ and $m_{2}$. The detailed BPAs are:

$$\begin{array}{c} m_{1}\left(x_{1}\right)=\frac{1}{4},m_{1}\left(x_{2}\right)=\frac{1}{3},m_{1}\left(x_{3}\right)=\frac{1}{6},m_{1}\left(x_{1},x_{2},x_{3}\right)=\frac{1}{6},m_{1}\left(x_{4}\right)=\frac{1}{12},\\ m_{2}\left(x_{1}\right)=\frac{1}{4},m_{2}\left(x_{2}\right)=\frac{1}{3},m_{2}\left(x_{3}\right)=\frac{1}{6},m_{2}\left(x_{1},x_{2}\right)=\frac{1}{6},m_{2}\left(x_{4}\right)=\frac{1}{12}. \end{array}$$

The corresponding $H_{PQ}\left(m_{1}\right)$ and $H_{PQ}\left(m_{2}\right)$ are calculated as follows:

$$\begin{array}{c} Pl_{m_{1}}\left(x_{1}\right)=\frac{5}{12},Pl_{m_{1}}\left(x_{2}\right)=\frac{1}{2},Pl_{m_{1}}\left(x_{3}\right)=\frac{1}{3},Pl_{m_{1}}\left(x_{4}\right)=\frac{1}{12},\\ Pl_{m_{2}}\left(x_{1}\right)=\frac{5}{12},Pl_{m_{2}}\left(x_{2}\right)=\frac{1}{2},Pl_{m_{2}}\left(x_{3}\right)=\frac{1}{6},Pl_{m_{2}}\left(x_{4}\right)=\frac{1}{12},\\ Pt_{m_{1}}\left(x_{1}\right)=\frac{5}{16},Pt_{m_{1}}\left(x_{2}\right)=\frac{6}{16},Pt_{m_{1}}\left(x_{3}\right)=\frac{4}{16},Pt_{m_{1}}\left(x_{4}\right)=\frac{1}{16},\\ Pt_{m_{2}}\left(x_{1}\right)=\frac{5}{14},Pt_{m_{2}}\left(x_{2}\right)=\frac{6}{14},Pt_{m_{2}}\left(x_{3}\right)=\frac{2}{14},Pt_{m_{2}}\left(x_{4}\right)=\frac{1}{14},\\ H_{PQ}\left(m_{1}\right)=\frac{1}{4}\times \log\left(\frac{1}{5/16}\right)+\frac{1}{3}\times \log\left(\frac{1}{6/16}\right)+\frac{1}{6}\times \log\left(\frac{1}{4/16}\right)+\ldots \\ \ldots +\frac{1}{6}\times \log\left(\frac{3}{15/16}\right)+\frac{1}{12}\times \log\left(\frac{1}{1/16}\right)=1.8375,\\ H_{PQ}\left(m_{2}\right)=\frac{1}{4}\times \log\left(\frac{1}{5/14}\right)+\frac{1}{3}\times \log\left(\frac{1}{6/14}\right)+\frac{1}{6}\times \log\left(\frac{1}{2/14}\right)+\ldots \\ \ldots +\frac{1}{6}\times \log\left(\frac{2}{13/14}\right)+\frac{1}{12}\times \log\left(\frac{1}{1/14}\right)=1.7485. \end{array}$$

It can be seen from the results of $H_{PQ}\left(m_{1}\right)$ and $H_{PQ}\left(m_{2}\right)$, the belief entropy of $m_{1}$ is larger than the $m_{2}$. This is logical because the $m_{1}\left(x_{1},x_{2},x_{3}\right)=\frac{1}{6}$ has one more single element than $m_{2}\left(x_{1},x_{2}\right)=\frac{1}{6}$, which implies that the $m_{1}\left(x_{1},x_{2},x_{3}\right)$ contains more information. Thus, the $m_{1}$ should be more uncertain.

### 5.5. Example 5

Consider a target recognition problem in [60]. Target detection results provided by two independent sensors. Let *A*, *B*, *C*, and *D* be the potential target types. The results are represented by BPAs shown as follows.

$$\begin{array}{c} m_{1}\left(A,B\right)=0.4,m_{1}\left(C,D\right)=0.6,\\ m_{2}\left(A,C\right)=0.4,m_{2}\left(B,C\right)=0.6. \end{array}$$

Then the corresponding uncertainty measure with $H_{deng}\left(m\right)$, $H_{pd}\left(m\right)$ and $H_{PQ}\left(m\right)$ are calculated as:

$$\begin{array}{c} Bel_{m_{1}}\left(A,B\right)=0.4,Pl_{m_{1}}\left(A,B\right)=0.4,Bel_{m_{1}}\left(C,D\right)=0.6,Pl_{m_{1}}\left(C,D\right)=0.6,\\ Bel_{m_{2}}\left(A,C\right)=0.4,Pl_{m_{2}}\left(A,C\right)=1.0,Bel_{m_{2}}\left(B,C\right)=0.6,Pl_{m_{2}}\left(B,C\right)=1.0,\\ H_{d}\left(m_{1}\right)=0.4\log\frac{2^{\left|2\right|}-1}{0.4}+0.6\log\frac{2^{\left|2\right|}-1}{0.6}=2.5559,\\ H_{d}\left(m_{2}\right)=0.4\log\frac{2^{\left|2\right|}-1}{0.4}+0.6\log\frac{2^{\left|2\right|}-1}{0.6}=2.5559,\\ H_{pd}\left(m_{1}\right)=\frac{0.4+0.4}{2}\log\frac{2^{\left|2\right|}-1}{(0.4+0.4)/2}+\frac{0.6+0.6}{2}\log\frac{2^{\left|2\right|}-1}{(0.6+0.6)/2}=2.5559,\\ H_{pd}\left(m_{2}\right)=\frac{0.4+1.0}{2}\log\frac{2^{\left|2\right|}-1}{(0.4+1.0)/2}+\frac{0.6+1.0}{2}\log\frac{2^{\left|2\right|}-1}{(0.6+1.0)/2}=2.9952,\\ Pl_{m_{1}}\left(A\right)=0.4,Pl_{m_{1}}\left(B\right)=0.4,Pl_{m_{1}}\left(C\right)=0.6,Pl_{m_{1}}\left(D\right)=0.6,\\ Pl_{m_{2}}\left(A\right)=0.4,Pl_{m_{2}}\left(B\right)=0.6,Pl_{m_{2}}\left(C\right)=1.0,\\ Pt_{m_{1}}\left(A\right)=0.2,Pt_{m_{1}}\left(B\right)=0.2,Pt_{m_{1}}\left(C\right)=0.3,Pt_{m_{1}}\left(D\right)=0.3,\\ Pt_{m_{2}}\left(A\right)=0.2,Pt_{m_{2}}\left(B\right)=0.3,Pt_{m_{2}}\left(C\right)=0.5,\\ H_{PQ}\left(m_{1}\right)=0.4\times \log\frac{2}{0.4}+0.6\times \log\frac{2}{0.6}=1.9710,\\ H_{PQ}\left(m_{2}\right)=0.4\times \log\frac{2}{0.7}+0.6\times \log\frac{2}{0.8}=1.3390. \end{array}$$

Though the two BPAs have the same value, the BPA $m_{1}$ has four potential targets, namely *A*, *B*, *C*, *D*, while the BPA $m_{2}$ has just three potential targets, namely *A*, *B*, *C*. As verified in [60], it is intuitively expected that $m_{1}$ has a larger uncertainty than $m_{2}$. According to the above calculation results, the $H_{deng}\left(m\right)$ illustrates that the two BPAs have the same uncertainty, and the $H_{pd}\left(m\right)$ suggests that the $m_{2}$ has a larger uncertainty. Therefore, both $H_{deng}\left(m\right)$ and $H_{pd}\left(m\right)$ are unable to reflect the prospective difference. The proposed belief entropy can effectively quantify this divergence by considering not only the information contained in each focal element but also the mutual support degree among different focal elements. Therefore, it is safe to say that the capability of the proposed belief entropy $H_{PQ}\left(m\right)$ is unavailable in the $H_{deng}\left(m\right)$ and $H_{pd}\left(m\right)$.

### 5.6. Example 6

Let $\Theta =\left\{x_{1},x_{2},x_{3},x_{4},x_{5},x_{6}\right\}$ be the FOD. Two BPAs are given as follows.

$$\begin{array}{c} m_{1}\left(x_{1},x_{2}\right)=\frac{1}{3},m_{1}\left(x_{3},x_{4}\right)=\frac{1}{3},m_{1}\left(x_{5},x_{6}\right)=\frac{1}{3},\\ m_{2}\left(x_{1},x_{2},x_{3}\right)=\frac{1}{2},m_{2}\left(x_{4},x_{5},x_{6}\right)=\frac{1}{2}. \end{array}$$

According to the Jiroušek-Shenoy entropy $H_{JS}\left(m\right)$ in Equation (23) and the proposed belief entropy $H_{PQ}\left(m\right)$ in Equation (24), both kinds of entropy consists of the discord uncertainty measure and the non-specificity uncertainty measure. Then the $H_{JS}\left(m\right)$ and $H_{PQ}\left(m\right)$ are calculated as follows.

$$\begin{array}{c} \begin{array}{c} Pl_{m_{1}}\left(x_{1}\right)=\frac{1}{3},Pl_{m_{1}}\left(x_{2}\right)=\frac{1}{3},Pl_{m_{1}}\left(x_{3}\right)=\frac{1}{3},Pl_{m_{1}}\left(x_{4}\right)=\frac{1}{3},Pl_{m_{1}}\left(x_{5}\right)=\frac{1}{3},Pl_{m_{1}}\left(x_{6}\right)=\frac{1}{3},\\ Pl_{m_{2}}\left(x_{1}\right)=\frac{1}{2},Pl_{m_{2}}\left(x_{2}\right)=\frac{1}{2},Pl_{m_{2}}\left(x_{3}\right)=\frac{1}{2},Pl_{m_{2}}\left(x_{4}\right)=\frac{1}{2},Pl_{m_{2}}\left(x_{5}\right)=\frac{1}{2},Pl_{m_{2}}\left(x_{6}\right)=\frac{1}{2},\\ Pt_{m_{1}}\left(x_{1}\right)=\frac{1}{6},Pt_{m_{1}}\left(x_{2}\right)=\frac{1}{6},Pt_{m_{1}}\left(x_{3}\right)=\frac{1}{6},Pt_{m_{1}}\left(x_{4}\right)=\frac{1}{6},Pt_{m_{1}}\left(x_{5}\right)=\frac{1}{6},Pt_{m_{1}}\left(x_{6}\right)=\frac{1}{6},\\ Pt_{m_{2}}\left(x_{1}\right)=\frac{1}{6},Pl_{m_{2}}\left(x_{2}\right)=\frac{1}{6},Pl_{m_{2}}\left(x_{3}\right)=\frac{1}{6},Pl_{m_{2}}\left(x_{4}\right)=\frac{1}{6},Pl_{m_{2}}\left(x_{5}\right)=\frac{1}{6},Pl_{m_{2}}\left(x_{6}\right)=\frac{1}{6},\\ H_{JS}\left(m_{1}\right)=H_{JS}^{dis}\left(m_{1}\right)+H_{JS}^{nos-spe}\left(m_{1}\right)=6\times \frac{1}{6}\times \log\left(\frac{1}{1/6}\right)+3\times \frac{1}{3}\times \log\left(2\right)=3.5850,\\ H_{JS}\left(m_{2}\right)=H_{JS}^{dis}\left(m_{2}\right)+H_{JS}^{nos-spe}\left(m_{2}\right)=6\times \frac{1}{6}\times \log\left(\frac{1}{1/6}\right)+2\times \frac{1}{2}\times \log(3)=4.1699,\\ H_{PQ}\left(m_{1}\right)=H_{PQ}^{dis}\left(m_{1}\right)+H_{PQ}^{nos-spe}\left(m_{1}\right)=3\times \frac{1}{3}\times \log\left(\frac{1}{1/3}\right)+3\times \frac{1}{3}\times \log\left(2\right)=2.5850,\\ H_{PQ}\left(m_{2}\right)=H_{PQ}^{dis}\left(m_{2}\right)+H_{PQ}^{nos-spe}\left(m_{2}\right)=2\times \frac{1}{2}\times \log\left(\frac{1}{1/2}\right)+2\times \frac{1}{2}\times \log\left(3\right)=2.5850. \end{array} \end{array}$$

The results calculated by the $H_{JS}^{dis}\left(m_{1}\right)$ and $H_{JS}^{dis}\left(m_{2}\right)$ are the same, which are equal to log(6). This outcome is counterintuitive. The BPAs in $m_{1}$ are completely different from that in $m_{2}$, thus the $H_{JS}^{dis}\left(m\right)$ and $H_{JS}^{non-spe}\left(m\right)$ of $m_{1}$ are expected to be distinguished from those ones of $m_{2}$. However, only the $H_{JS}^{non-spe}\left(m_{1}\right)$ and $H_{JS}^{non-spe}\left(m_{2}\right)$ are different. The reason for this situation is that the discord uncertainty measure $H_{JS}^{dis}\left(m\right)$ in $H_{JS}\left(m\right)$ overly concerns the conflict involved in single elements and ignores the information contained in the original BPAs. The discord uncertainty measure $H_{PQ}^{dis}\left(m\right)$ in $H_{PQ}\left(m\right)$ combines the original BPAs with the probability distribution of single elements included in the BPA can better resolve the limitations. In short, this example indicates the effectiveness for measuring the discord uncertainty of the proposed belief entropy.

### 5.7. Example 7

Let Θ=(1,2,…,14,15) be a FOD with 15 elements. The mass functions of Θ is denoted as:

$$\begin{array}{c} m(3,4,5)=0.05,\;m(7)=0.05,\;m(A)=0.8,\;m(\Theta )=0.1 \end{array}$$

**Figure 1:**
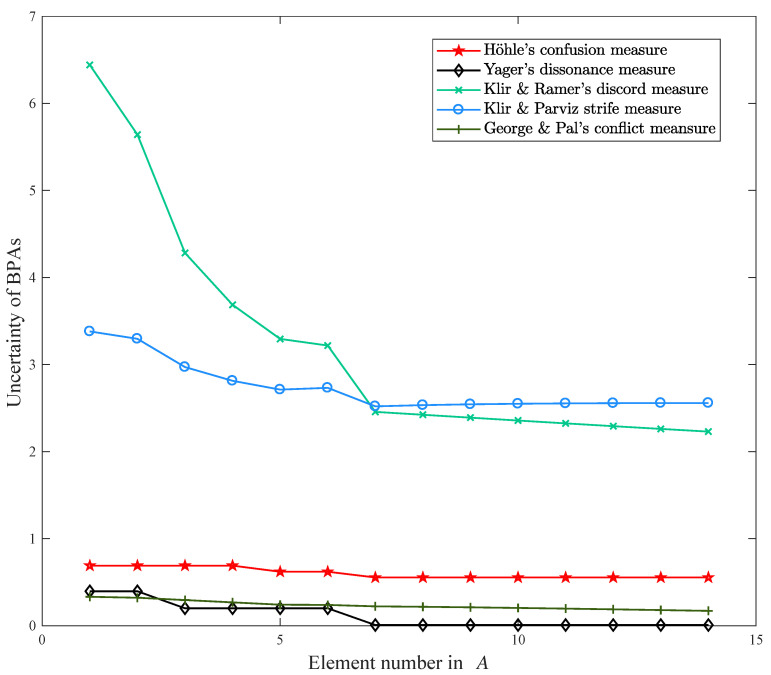
Results comparison of $M_{2}$, $M_{3}$, $M_{4}$, $M_{5}$, and $M_{6}$ in DST (Dempster-Shafer evidence theory).

**Figure 2:**
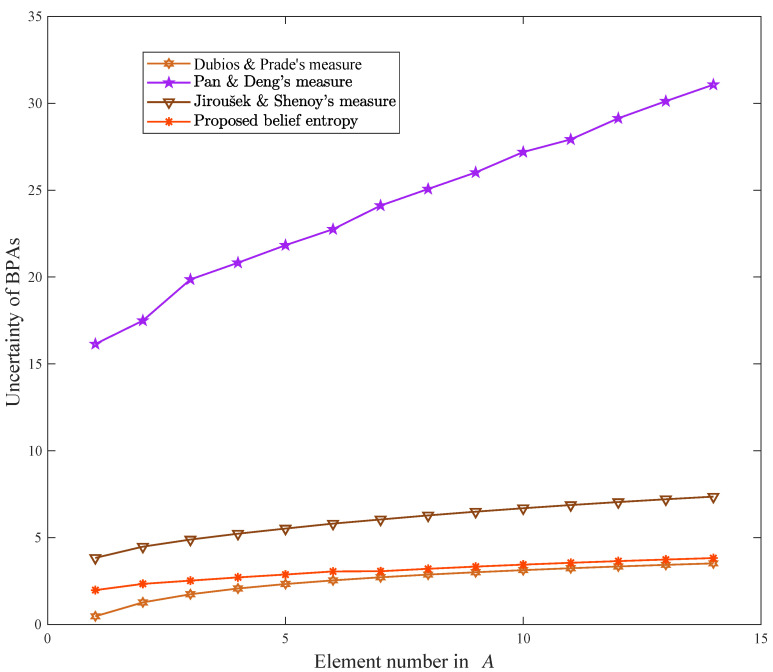
Results comparison of $M_{1}$, $M_{7}$, $M_{8}$, and $M_{9}$ in DST.

The proposition *A* is a variable subset of $2^{\Theta }$ with the number of single elements changing from 1 to 14. To verify the merit and effectiveness of the proposed belief entropy, eight uncertainty measures listed in Table 1 are selected for comparison, including Dubois and Prade’s weighted Hartley entropy [33], Höhle’s confusion uncertainty measure [34], Yager’s dissonance uncertainty measure [35], Klir and Ramer’s discord uncertainty measure [36], Klir and Parviz’s strife uncertainty measure [37], George and Pal’s conflict uncertainty measure [61], Pan and Deng’s uncertainty measure [40], Jiroušek and Shenoy’s uncertainty measure [41]. The experimental results are shown in Table 2. The Höhle’s confusion uncertainty measure ($M_{2}$), Yager’s dissonance uncertainty measure ($M_{3}$), Klir and Ramer’s discord uncertainty measure ($M_{4}$), Klir and Parviz’s strife uncertainty measure ($M_{5}$), and George and Pal’s conflict uncertainty measure ($M_{6}$) are plotted in Figure 1. The Dubois and Prade’s weighted Hartley entropy ($M_{1}$), Pan and Deng’s uncertainty measure ($M_{7}$), Jiroušek and Shenoy’s uncertainty measure ($M_{8}$), and proposed belief entropy ($M_{9}$) are plotted in Figure 2.

As shown in Figure 1, it is obvious that the uncertain degree measured by the George and Pal’s conflict measure is almost unchanged when the element number increase in proposition *A*. Similarly, the Höhle’s confusion uncertainty measure and Yager’s dissonance uncertainty measure have the same situation to reflect the variation on uncertain degree in this case. Thus, these three uncertainty measures cannot detect the change in proposition *A*. Although the uncertainty degrees obtained by the Klir and Ramer’s discord uncertainty measure and Klir and Parviz’s strife uncertainty measure change with the growth of element number in *A*, the variation trends of both methods are contrary to expectation that the uncertainty degree increases with the augment of the element number in *A*. These methods only measure the discord uncertainty of the BPAs, but ignore the non-specificity uncertainty of the BPAs. Besides, from the Table 2, we can find that the Yager’s dissonance uncertainty measure has the minimum uncertainty degree. This is because this method uses the plausibility function to measure the discord uncertainty. The plausibility function contains all the support degree to the single events from other propositions, which could lead to information redundant and uncertainty reducing incorrectly. To sum up, the uncertainty degree obtained by George and Pal’s method, Höhle’s method, Yager’s method, Klir and Ramer’s method, and Klir and Parviz’s method are unreasonable and counterintuitive, which means these methods cannot measure the uncertainty in this case aright.

From Figure 2, it can be seen that the uncertainty degrees measured by Dubois and Prade’s weighted Hartley entropy, Pan and Deng’s uncertainty measure, Jiroušek and Shenoy’s uncertainty measure, and the proposed belief entropy are increasing visibly with the rising of the element number in *A*. These methods consider not only the discord uncertainty but also the non-specificity uncertainty. Furthermore, Pan and Deng’s uncertainty measure is the largest among all the methods in Table 2. This is understandable. The non-specificity uncertainty measure in Pan and Deng’s method is exponential, while the others are linear. As the number of elements in *A* increases, the uncertainty degree of Pan and Deng’s method increases faster than the other methods. Non-specificity uncertainty measure using exponential form may cause the possible uncertainty degree from the discord part to be significantly smaller than the ones from the non-specificity part. Additionally, Jiroušek and Shenoy’s uncertainty measure is larger than the proposed belief entropy. Compared to the Jiroušek and Shenoy’s uncertainty measure, which uses the probability distribution of single element obtained by plausibility transformation to measure the discord uncertainty, the proposed belief entropy measure that one by using the information of each mass function and the single element each BPA contains. The redundant information is removed, and the possible values of discord uncertainty is decreased notably in the proposed method. More importantly, except for the proposed method, the other three uncertainty measures have shortcomings. The Dubois and Prade’s weighted Hartley entropy does not consider the discord uncertainty of BPAs. The Pan and Deng’s uncertainty measure cannot measure accurately two similar BPAs in Section 5.5. The discord uncertainty measure of Jiroušek and Shenoy’s uncertainty measure is irrational in Section 5.6. Thus, the proposed belief entropy is the only effective approach for uncertainty measure among these given methods in this case. Therefore, the proposed belief entropy, which considers the information contained in BPAs and single elements, is reasonable and effective for uncertainty measure in Dempster-Shafer framework.

## 6. Conclusions

How to measure the uncertainty of BPA in the framework of DST is an open issue. In this study, the main contribution is that a new belief entropy is proposed to quantify the uncertainty of BPA. The proposed belief entropy is comprised of the discord uncertainty measurement and the non-specificity uncertainty measurement. In particular, in the discord uncertainty measure component, the idea of probability interval and conversion BPA to probability using the plausibility transformation are combined. The new method takes advantage of the information of not only the BPAs, but also the total support degree of the single events contained in the BPAs. By addressing appropriate information in a BPA, which means less information loss and less information redundancy, the proposed belief entropy could measure the uncertainty of BPA efficiently. In addition, the proposed belief entropy could satisfy six desired properties of consistency with DST semantics, non-negativity, set consistency, probability consistency, additivity, and monotonicity. The results of numerical experiments demonstrate that the proposed belief entropy can be more effective and accurate when compared to the existing uncertainty measures in the framework of DST. Future work of this study will be focused on extending the proposed method to open-world assumptions and applying it to solve problems in real applications.

## Figures and Tables

**Table 1:** An overview of the properties of existing belief entropies and the proposed method.

Definition	Cons.w DST	Non-neg	Max. ent	Monoton	Prob. cons	Add	Subadd	Range	Set. cons
Höhle	yes	no	no	no	yes	yes	no	yes	no
Smets	yes	no	no	no	no	yes	no	yes	no
Yager	yes	no	no	no	yes	yes	no	yes	no
Nguyen	yes	no	no	no	yes	yes	no	yes	no
Dubois-Prade	yes	no	yes	yes	no	yes	yes	yes	yes
Klir-Ramer	yes	yes	no	yes	yes	yes	no	no	yes
Klir-Parviz	yes	yes	no	yes	yes	yes	no	no	yes
Pal et al.	yes	yes	no	yes	yes	yes	no	no	yes
George-Pal	yes	no	no	no	no	no	no	no	yes
Maeda-Ichihashi	no	yes	yes	yes	yes	yes	yes	no	yes
Harmanec-Klir	no	yes	no	yes	yes	yes	yes	no	no
Abellán-Moral	no	yes	yes	yes	yes	yes	yes	no	yes
Jousselme et al.	no	yes	no	yes	yes	yes	no	yes	yes
Pouly et al.	no	yes	no	yes	yes	yes	no	no	yes
Jiroušek-Shenoy	yes	yes	yes	yes	yes	yes	no	no	no
Deng	yes	yes	no	yes	yes	no	no	no	no
Pan-Deng	yes	yes	no	yes	yes	no	no	no	no
Proposed method	yes	yes	no	yes	yes	yes	no	no	yes

**Table 2:** The value of different uncertainty measures.

Cases	$M_{1}$	$M_{2}$	$M_{3}$	$M_{4}$	$M_{5}$	$M_{6}$	$M_{7}$	$M_{8}$	$M_{9}$
A = $\left\{1\right\}$	0.4699	0.6897	0.3953	6.4419	3.3804	0.3317	16.1443	3.8322	1.9757
A = $\left\{1,2\right\}$	1.2699	0.6897	0.3953	5.6419	3.2956	0.3210	17.4916	4.4789	2.3362
A = $\left\{1,2,3\right\}$	1.7379	0.6897	0.1997	4.2823	2.9709	0.2943	19.8608	4.8870	2.5232
A = $\left\{1,2,3,4\right\}$	2.0699	0.6897	0.1997	3.6863	2.8132	0.2677	20.8229	5.2250	2.7085
A = $\left\{1,2,3,4,5\right\}$	2.3275	0.6198	0.1997	3.2946	2.7121	0.2410	21.8314	5.5200	2.8749
A = $\left\{1,2,\ldots ,6\right\}$	2.5379	0.6198	0.1997	3.2184	2.7322	0.2383	22.7521	5.8059	3.0516
A = $\left\{1,2,\ldots ,7\right\}$	2.7158	0.5538	0.0074	2.4562	2.5198	0.2220	24.1131	6.0425	3.0647
A = $\left\{1,2,\ldots ,8\right\}$	2.8699	0.5538	0.0074	2.4230	2.5336	0.2170	25.0685	6.2772	3.2042
A = $\left\{1,2,\ldots ,9\right\}$	3.0059	0.5538	0.0074	2.3898	2.5431	0.2108	26.0212	6.4921	3.3300
A = $\left\{1,2,\ldots ,10\right\}$	3.1275	0.5538	0.0074	2.3568	2.5494	0.2037	27.1947	6.6903	3.4445
A = $\left\{1,2,\ldots ,11\right\}$	3.2375	0.5538	0.0074	2.3241	2.5536	0.1959	27.9232	6.8743	3.5497
A = $\left\{1,2,\ldots ,12\right\}$	3.3379	0.5538	0.0074	2.2920	2.5562	0.1877	29.1370	7.0461	3.6469
A = $\left\{1,2,\ldots ,13\right\}$	3.4303	0.5538	0.0074	2.2605	2.5577	0.1791	30.1231	7.2071	3.7374
A = $\left\{1,2,\ldots ,14\right\}$	3.5158	0.5538	0.0074	2.2296	2.5582	0.1701	31.0732	7.3587	3.8219

$M_{1}$ is the Dubois and Prade’s weighted Hartley entropy; $M_{2}$ is the Höhle’s confusion uncertainty measure; $M_{3}$ is the Yager’s dissonance uncertainty measure; $M_{4}$ is the Klir and Ramer’s discord uncertainty measure; $M_{5}$ is the Klir and Parviz’s strife uncertainty measure; $M_{6}$ is the George and Pal’s conflict uncertainty measure; $M_{7}$ is the Pan and Deng’s uncertainty measure; $M_{8}$ is the Jiroušek and Shenoy’s uncertainty measure; $M_{9}$ is the proposed belief entropy.
